# Supplementary material for: Signal Transduction Pathway Activity in High-Grade, Serous Ovarian Carcinoma Reveals a More Favorable Prognosis in Tumors with Low PI3K and High NF-κB Pathway Activity: A Novel Approach to a Long-Standing Enigma
Source: Cancers (Basel). 2020 Sep 18;12(9):2660. doi: 10.3390/cancers12092660 (PMC7564278; doi:10.3390/cancers12092660)
Supplement: Supplementary file 1 [file cancers-12-02660-s001.pdf]

Supplementary Materials: Signal Transduction Pathway Activity in High-Grade, Serous Ovarian Carcinoma Reveals a More Favorable Prognosis in Tumors with Low PI3K and High NF-κB Pathway Activity: A Novel Approach to A Long-Standing Enigma

Laura van Lieshout, Anja van de Stolpe, Phyllis van der Ploeg, David Bowtell, Joanne de Hullu and Jurgen Piek

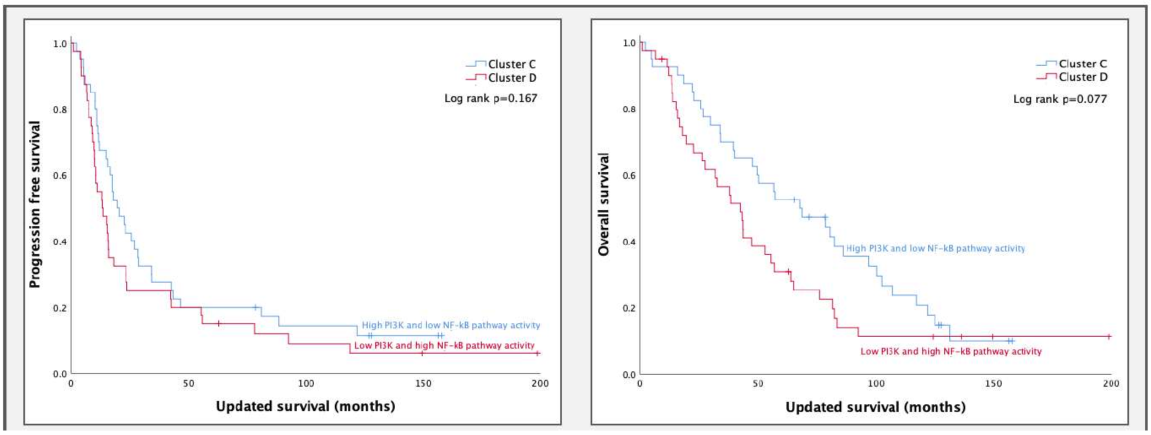

Figure S1. Progression free survival (PFS) and overall survival (OS) of the newly formed clusters of the grade 2 serous carcinoma samples (n = 80) in dataset GSE9891 using additional follow-up data provided by the Australian Ovarian Cancer Study.

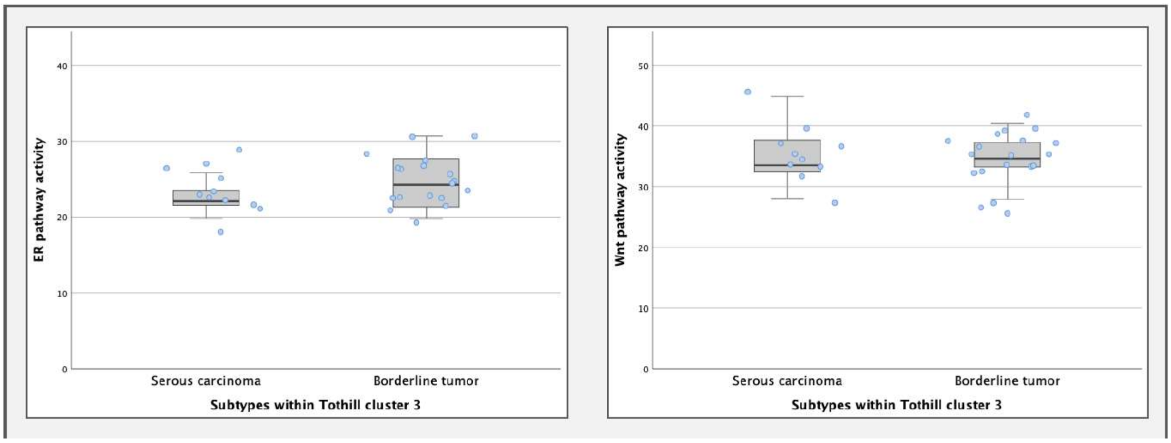

Figure S2. Boxplots for pathway activity per subtype within Tothill cluster 3 with overlying dot plots representing individual samples.

© 2020 by the authors. Licensee MDPI, Basel, Switzerland. This article is an open access article distributed under the terms and conditions of the Creative Commons Attribution (CC BY) license (http://creativecommons.org/licenses/by/4.0/).
